# Protein kinase CK2 activation is required for transforming growth factor β‐induced epithelial–mesenchymal transition

**DOI:** 10.1002/1878-0261.12378

**Published:** 2018-09-21

**Authors:** Seongrak Kim, Sunyoung Ham, Kyungmi Yang, Kunhong Kim

**Affiliations:** ^1^ Department of Biochemistry and Molecular Biology Yonsei University College of Medicine Seoul Korea; ^2^ Integrated Genomic Research Center for Metabolic Regulation Seoul Korea; ^3^ Quality Evaluation Team Samsung Bioepis Incheon Korea

**Keywords:** carboxyl terminus of Hsc70‐interacting protein, Epithelial–mesenchymal transition, protein kinase CK2, TGFβ, WW domain containing E3 ubiquitin protein ligase 1

## Abstract

Transforming growth factor β (TGFβ) is overexpressed in advanced cancers and promotes tumorigenesis by inducing epithelial–mesenchymal transition (EMT), which enhances invasiveness and metastasis. Although we previously reported that EMT could be induced by increasing CK2 activity alone, it is not known whether CK2 also plays an essential role in TGFβ‐induced EMT. Therefore, in the present study, we investigated whether TGFβ signaling could activate CK2 and, if so, whether such activation is required for TGFβ‐induced EMT. We found that CK2 is activated by TGFβ treatment, and that activity peaks at 48 h after treatment. CK2 activation is dependent on TGFβ receptor (TGFBR) I kinase activity, but independent of SMAD4. Inhibition of CK2 activation through the use of either a CK2 inhibitor or shRNA against *CSNK2A1* inhibited TGFβ‐induced EMT. TGFβ signaling decreased CK2β but did not affect CK2α protein levels, resulting in a quantitative imbalance between the catalytic α and regulatory β subunits, thereby increasing CK2 activity. The decrease in CK2β expression was dependent on TGFBRI kinase activity and the ubiquitin–proteasome pathway. The E3 ubiquitin ligases responsible for TGFβ‐induced CK2β degradation were found to be CHIP and WWP1. Okadaic acid (OA) pretreatment protected CK2β from TGFβ‐induced degradation, suggesting that dephosphorylation of CK2β by an OA‐sensitive phosphatase might be required for CK2 activation in TGFβ‐induced EMT. Collectively, our results suggest CK2 as a therapeutic target for the prevention of EMT and metastasis of cancers.

**Abbreviations**
CHIP: carboxyl terminus of Hsc70‐interacting protein
CK2: protein kinase CK2
CKD: *CSNK2A1* knockdown
EMT: epithelial–mesenchymal transition
HEK: human embryonic kidney; NSCLC; non‐small cell lung cancer
OA: okadaic acid
SKD: *SMAD4‐*knockdown
TGFBRII: TGFβ type II receptor
TGFBRI: TGFβ type I receptor
TGFβ: transforming growth factor β
WWP1: WW domain containing E3 ubiquitin protein ligase 1

## 1 Introduction

Transforming growth factor β (TGFβ) is a potent pleiotropic cytokine that regulates cell growth/differentiation, cell motility, extracellular matrix production, angiogenesis and cellular immune responses (Derynck *et al*., 2001; Dumont and Arteaga, 2003). TGFβ has three isoforms, TGFβ1, TGFβ2 and TGFβ3, whose specific roles have been revealed by knockout mouse studies (Dickson *et al*., 1995; Proetzel *et al*., 1995; Sanford *et al*., 1997). TGFβ signaling is mediated through SMAD and non‐SMAD pathways to regulate transcription, translation, microRNA biogenesis, protein synthesis and post‐translational modifications (Frey and Mulder, 1997; Hata and Davis, 2009; Hussey *et al*., 2011; Mu *et al*., 2012; Park *et al*., 2004; Yu *et al*., 2002). TGFβ binds to TGFβ type II receptor (TGFBRII) containing a constitutively active serine/threonine kinase domain. Ligand binding induces receptor complex formation between TGFBRII and TGFβ type I receptor (TGFBRI), inducing the phosphorylation and activation of TGFBRI by TGFBRII. In the canonical SMAD pathway, phosphorylated and activated TGFBRI recruits and phosphorylates receptor‐regulated SMAD (R‐SMAD) (Shi and Massague, 2003). Phosphorylated R‐SMADs form complexes with SMAD4 and translocate into the nucleus, where they activate or repress the expression of TGFβ‐responsive genes in a cell type‐ and context‐dependent manner (Koinuma *et al*., 2009; Massague, 2008). In the non‐SMAD pathways, TGFβ activates p38 MAPK, p42/p44 MAPK, c‐Src, m‐TOR, RhoA, RAS, PI3K/Akt, protein phosphatase 2A (PP2A)/p70S6K and JNK‐MAPK (Hong *et al*., 2011; Kang *et al*., 2009; Mu *et al*., 2012).

TGFβ inhibits cell cycle progression and proliferation in benign cells in the early stages of tumorigenesis (Principe *et al*., 2014). TGFβ overexpression is demonstrated in many cancers (Bruna *et al*., 2007; Chod *et al*., 2008; Labidi *et al*., 2010; Langenskiold *et al*., 2008; Shariat *et al*., 2008) and is related to a poor prognosis (Tsushima *et al*., 1996; Wikstrom *et al*., 1998). It induces tumor progression, including enhancement of tumor cell proliferation, invasion and metastasis (Akhurst and Derynck, 2001; Inman, 2011; Langenskiold *et al*., 2008; Massague, 2008; Padua and Massague, 2009; Pasche, 2001). The three most common mechanisms underlying TGFβ‐mediated tumor progression are epithelial–mesenchymal transition (EMT), increased invasiveness and metastasis, and immunosuppression (Haque and Morris, 2017).

EMT is a biological process in which cells lose epithelial characteristics; however, they also acquire mesenchymal characteristics through multiple biochemical changes (Kalluri and Neilson, 2003). The transitioned cells are characterized by loss of epithelial cell polarity, cell–cell junction disassembly and increased cell motility (Ikenouchi *et al*., 2003). EMT occurs in many biological processes, such as implantation, embryogenesis, organ development, wound healing, tissue regeneration, organ fibrosis and tumor progression (Kalluri and Weinberg, 2009). The E‐ to N‐cadherin switch, often occurring during EMT, is the replacement of E‐cadherin expression with N‐cadherin expression (Cavallaro *et al*., 2002; Christofori, 2003; Hsu *et al*., 1996; Li *et al*., 2001; Scott and Cassidy, 1998; Tang *et al*., 1994) and a molecular hallmark of EMT (Kalluri and Weinberg, 2009). Transcriptional E‐cadherin repression is a major molecular mechanism underlying E‐cadherin expression loss during the cadherin switch (Thiery and Sleeman, 2006). E‐cadherin transcriptional repressors, whose expression or activity is regulated by TGFβ signaling, include the Snail superfamily of zinc‐finger transcriptional repressors, Snail1 (Batlle *et al*., 2000; Cano *et al*., 2000) and Snail2 (also called Slug) (Bolos *et al*., 2003; Hajra *et al*., 2002), the ZEB family of transcription factors, ZEB1 (also called TCF8 and δEF1) and ZEB2 [also called ZFXH1B and SMAD‐interacting protein 1 (SIP1)] (Comijn *et al*., 2001; Eger *et al*., 2005), bHLH factors, Twist1, E47 (also called TCF3) and TCF4 (also called E2‐2)(Perez‐Moreno *et al*., 2001; Yang *et al*., 2004).

Protein kinase CK2 is a constitutively active, growth factor‐independent serine/threonine‐protein kinase with key roles in cell cycle regulation, cellular differentiation, proliferation and apoptosis regulation (Ahmad *et al*., 2005; Shin *et al*., 2005; Song *et al*., 2000). Changes in CK2 expression or activity have been reported in many cancers (Kim *et al*., 2007; Landesman‐Bollag *et al*., 2001; Scaglioni *et al*., 2006; Shin *et al*., 2005) and overexpression of the catalytic subunit of CK2 can induce tumorigenesis (Landesman‐Bollag *et al*., 2001). CK2 is also a positive regulator of Wnt signaling, which is important for metastasis (Seldin *et al*., 2005). Recently, we reported that an increase in CK2 activity induced the E‐ to N‐cadherin switch (Ko *et al*., 2012). Although an increase in CK2 activity could induce the E‐to N‐cadherin switch, it is not known whether CK2 plays a role in TGFβ‐induced EMT. Because it is well known that TGFβ induces EMT, the present study aimed to investigate whether TGFβ signaling could activate CK2 and also whether the activation was essential for TGFβ‐induced EMT.

## 2 Materials and methods

### 2.1 Cell culture, reagents and plasmid

A human non‐small cell lung cancer (NSCLC) cell line, A549 was cultured in Roswell Park Memorial Institute 1640 medium (Gibco Laboratories, Gaithersburg, MD, USA), supplemented with 10% fetal bovine serum (Gibco), 100 μg·mL^−1^ streptomycin (Gibco), and 100 U·mL^−1^ penicillin (Gibco). Human embryonic kidney (HEK) 293 cells were cultured in Dulbecco's modified Eagle's medium (Gibco). All cells were cultured at 37 °C in 5% CO_2_. The CK2 inhibitor, emodin (Sigma‐Aldrich, St Louis, MO, USA), and the reversible cell‐permeable proteasome inhibitor, MG132 (Sigma‐Aldrich), were prepared in 20 mm stock with dimethylsulfoxide (Sigma‐Aldrich). The TGFBRI‐specific inhibitor, SB431542 (Sigma‐Aldrich), was prepared in 10 mm stock with dimethylsulfoxide. The protein phosphatase inhibitor, okadaic acid (OA; Sigma‐Aldrich), was prepared in 10 μm stock with dimethylsulfoxide. TGFβ (R&D Systems, Minneapolis, MN, USA) was prepared in 10 μg·mL^−1^ stock. pCMV5 TBRI‐His was a gift from Joan Massague (Addgene plasmid # 19161). pCMV5B‐TGFβ receptor I K232R was a gift from Jeff Wrana (Addgene plasmid # 11763). p3TP‐Lux (Wrana *et al*., 1992), containing the *plasminogen activator inhibitor‐1* (*PAI‐1*) gene TGFβ response element and three collagenase I AP‐1 repeats in front of luciferase, was a gift from Joan Massague & Jeff Wrana (Addgene plasmid # 11767). pCMV5B‐Flag‐Smurf1 wt was a gift from Jeff Wrana (Addgene plasmid # 11752), pCMV5B‐Flag‐Smurf2 wt was a gift from Jeff Wrana (Addgene plasmid # 11746), p4489 Flag‐betaTrCP was a gift from Peter Howley (Addgene plasmid # 10865), pcDNA3‐HA2‐ROC1 was a gift from Yue Xiong (Addgene plasmid # 19897) and pCI HA NEDD4 was a gift from Joan Massague (Addgene plasmid # 27002). pCMV‐Tag3B‐WWP1‐myc was kindly provided by Dr Ceshi Chen (Kunming Institute of Zoology). Flag‐tagged pCMV‐Tag2C‐WWP1 was kindly provided by Dr Hyeon Soo Kim (Lee *et al*., 2013).

### 2.2 Western blot analysis

Western blot analysis was performed as described previously (Ko *et al*., 2012). Blotted membranes were immunostained with antibodies specific for the following antigens: HA tag (Covance, New York, NY, USA); Myc tag, Smad2/3, phosphoSmad2/3 and carboxyl terminus of Hsc70‐interacting protein (CHIP; Cell Signaling, Danvers, MA, USA); Flag tag and β‐actin (Sigma‐Aldrich); 6 × His tag and CK2β (R&D Systems); E‐cadherin and N‐cadherin (Thermo Fisher Scientific, Rockford, IL, USA); WW domain containing E3 ubiquitin protein ligase 1 (WWP1) (ProteinTech Group, Inc., Chicago, IL, USA); CK2α (EMD Millipore, Burlington, MA, USA); and HDAC I, Smad4 and vimentin (Santa Cruz Biotechnology Inc., Santa Cruz, CA, USA). The secondary antibodies were goat anti‐rabbit IgG peroxidase, goat anti‐mouse IgG horseradish peroxidase (Thermo Fisher Scientific) and donkey anti‐sheep IgG horseradish peroxidase (R&D Systems). Signals were developed using Lumi‐Light Western Blotting Substrate (Roche Diagnostics, Indianapolis, IN, USA) in accordance with the manufacturer's instructions.

### 2.3 *In vitro* kinase assay

To evaluate intracellular CK2 activity, an *in vitro* kinase assay was performed as described previously with slight modification (Scaglioni *et al*., 2006). Bacterially expressed GST‐CS (CK2 Substrate; GST‐RRRDDDSDDD) (3 μg) was incubated with glutathione‐Sepharose 4B beads for 60 min, and washed twice with kinase buffer (4 mm Mops, pH 7.2, 5 mm β‐glycerophosphate, 1 mm EGTA, 200 μm sodium orthovanadate, and 200 μm dithiothreitol). The beads were incubated with 100 μg of cell lysates in a final volume of 50 μL of kinase reaction buffer (10 μL of 5 × kinase buffer, 10 μL magnesium/ATP cocktail [90 μL of 75 mm MgCl_2_/500 mm ATP and 10 μL (100 μCi) of [γ‐^32^P]‐ATP]) for 20 min at 30 °C. The reactions were stopped by washing twice with 1 × kinase buffer. The samples were resuspended with 30 μL of 2 × SDS/PAGE sample‐loading buffer, subjected to 12% SDS/PAGE, stained with Coomassie Brilliant Blue, and dried on Whatman paper (GE Healthcare Life Sciences, Little Chalfont, UK). ^32^P incorporation was detected by autoradiography.

### 2.4 shRNA and siRNA

shRNA‐mediated knockdown of *CSNK2A1* (Ko *et al*., 2012) *or SMAD4* was performed using the HuSH‐plasmid system (Origene Technologies Inc., Rockville, MD, USA). The shRNA sequences tested for *SMAD4* knockdown were: sequence #1: TTCAGGTGGCTGGTCGGAAAGGATTTCCT; sequence #2: GCAGCCATAGTGAA GGACTGTTGCAGATA; sequence #3: CCAACATTCCTGTGGCTTCCACAAGTC AG; and sequence #4: GTCAGGTGCCTTAGTGACCACGCGGTCTT. We validated all constructs individually and found that constructs #1 and #2 were effective for *SMAD4* knockdown. Subsequently, we used construct #1 for *SMAD4* knockdown. Mission® esiRNA human *STUB1* was purchased from Sigma‐Aldrich. Silencer® select pre‐designed siRNA targeting human *WWP1* (human; siRNA ID s21788) was purchased from Ambion (Thermo Fisher Scientific). The cells were transfected with siRNA using Lipofectamine® RNAiMAX (Invitrogen/Life Technologies, Carlsbad, CA, USA) in accordance with the manufacturer's instructions.

### 2.5 Dual‐luciferase reporter assay

The cells were seeded in six‐well plates and cotransfected with p3TP‐Lux and pRL‐TK using ViaFect™ (Promega Corp., Madison, WI, USA). Twenty‐four hours after transfection, the cells were treated with TGFβ for 24 h, washed with PBS and harvested. Cell lysates were prepared with 200 μL of Passive Lysis buffer (Promega). Aliquots (20 μL) of cleared lysate were analyzed for luciferase activity using a Dual‐luciferase® reporter assay system (Promega). The luciferase activity of p3TP‐Lux was normalized to that of pRL‐TK.

### 2.6 Cell fractionation

The cells were allowed to swell in buffer A comprising 10 mm Hepes (pH 7.9), 10 mm KCl, 0.1 mm EDTA, 1 mm dithiothreiltol, 1 mm phenylmethanesulfonyl fluoride, 1 × protease inhibitor cocktail and 1 mm sodium orthovanadate. The samples were adjusted to 0.6% Nonidet P‐40 (NP‐40), and vortexed vigorously for 10 s. Nuclei were pelleted by centrifugation at 10 000 × ***g*** for 30 s at 4 °C. The supernatants were collected and used as the cytoplasmic fraction. After washing the pellets with PBS, they were lysed in buffer C comprising 20 mm Hepes, pH 7.9, 0.4 m NaCl, 0.1 mm EDTA, 1 mm dithiothreitol, 1 mm phenylmethanesulfonyl fluoride, 1 × protease inhibitor cocktail and 1 mm sodium orthovanadate by sonication. The lysates were cleared by centrifugation at 10 000 × ***g*** for 20 min at 4 °C. The supernatants were collected and used as the nuclear fraction.

### 2.7 Immunoprecipitation

The cells were collected and lysed with 1 mL of immunoprecipitation lysis buffer (50 mm Tris‐HCl, pH 7.4, 150 mm NaCl, 0.5% NP‐40) with cOmplete™ protease inhibitor cocktail (Roche Diagnostics). The cell lysates were pre‐cleared and then incubated with the appropriate antibodies for 1 h at 4 °C. The antibody–protein complexes were precipitated with Protein A/G‐Sepharose beads (Santa Cruz Biotechnology Inc.), washed, and resuspended in 40 μL of SDS/PAGE loading buffer.

### 2.8 Site‐directed mutagenesis

To generate mutants of CK2β with the autophosphorylation sites (serine 2, 3, and 4) mutated to non‐phosphorylatable alanine residues or to phosphomimetic glutamic acids or to generate TGFBRI constitutively active (CA) mutant (threonine 204 is replaced with aspartic acid), mutagenesis was performed using a QuikChange site‐directed mutagenesis kit (Stratagene, Cedar Creek, TX, USA). All mutant constructs were confirmed by DNA sequencing. The mutagenic primer pairs used to generate mutants were: CK2β 3E (S2ES3ES4E): forward, 5′‐GACGTGAAGATGGAAGAAGAAGAGGAGGTGTCC‐3′; reverse, 5′‐GGACACCTCCTCTTCTTCTTCGATCTTCACGTC‐3′. CK2β 3A (S2A S3AS4A): forward, 5′‐GACGTGAAGATGGCAGCAGCAGAGGAGGTGTCC‐3′; reverse, 5′‐GGACACCTCCTCTGCTGCTGCCATCTTCACGTC‐3′. TGFBRI CA: forward, 5′‐GAACAATTGCGAGAGATATTGTGTTACAAG‐3′; reverse, 5′‐TCCGTA ACACAATATCTCTCGCAATTGTTC‐3′.

### 2.9 Cell migration assay

A cell migration assay was conducted using specific wound‐assay chambers purchased from ibidi GmbH (Munich, Germany). All experiments were performed in accordance with the manufacturer's instructions.

### 2.10 Statistical analysis

Statistical comparisons of groups were performed using Student's *t*‐test. *P* < 0.05 was considered statistically significant.

## 3 Results

### 3.1 CK2 activation was required for TGFβ‐induced EMT

**Figure 1:**
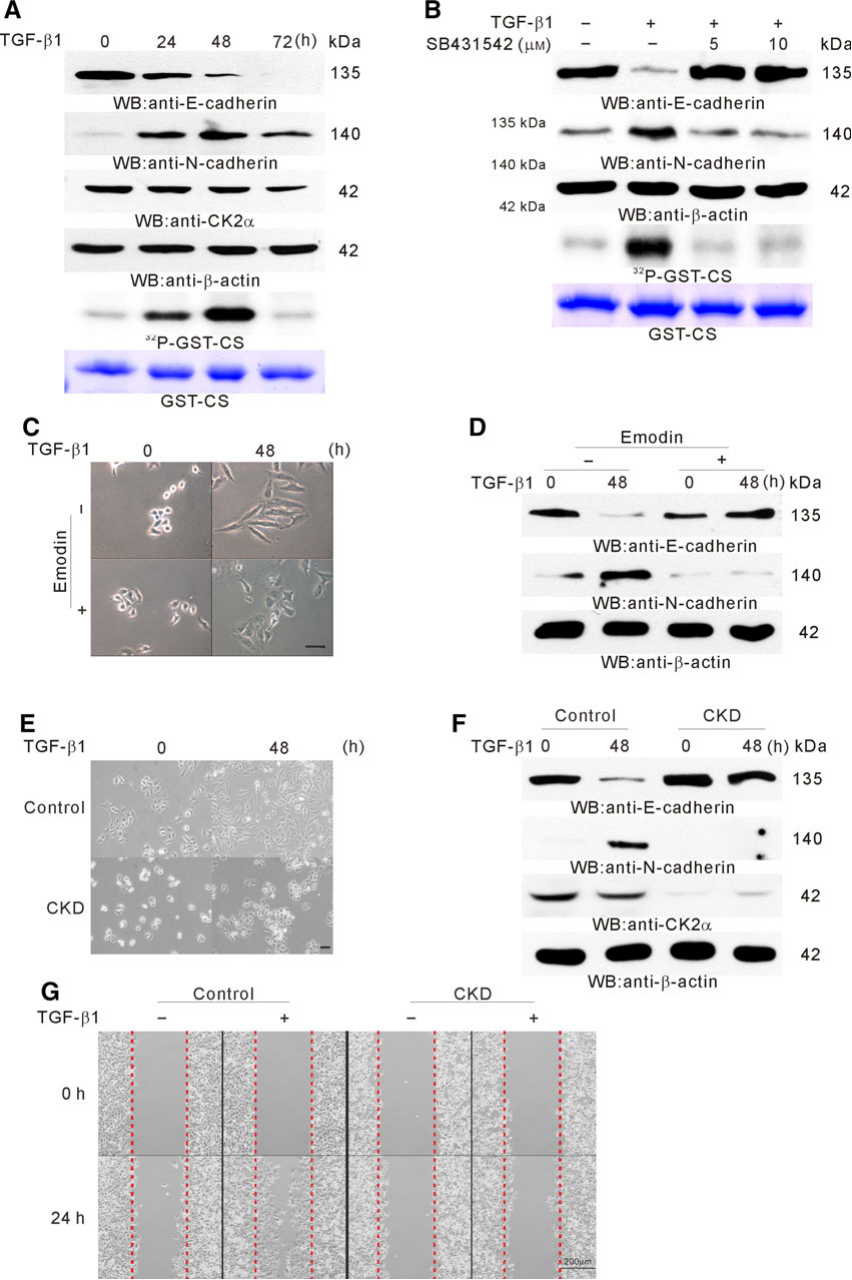
Requirement of CK2 activation in TGFβ‐induced EMT. (A) CK2 activation during TGFβ‐induced EMT. A549 cells were treated with TGFβ for the indicated time periods. Western blot analysis was performed with the indicated antibodies (top) and an *in vitro* kinase assay was performed using the same lysates (bottom). GST‐CS represents input GST‐CS stained with Coomassie Brilliant Blue. ^32^P‐GST‐CS represents phosphorylated GST‐CS. (B) Effect of TGFBRI kinase inhibitor on CK2 activation. A549 cells were pretreated or untreated with SB431542 (10 μm) for 12 h, and then with TGFβ for 48 h. Western blot analysis was performed with the indicated antibodies (top) and an *in vitro* kinase assay was performed (bottom). (C) Effect of a CK2 inhibitor on the morphology of TGFβ‐treated cells. A549 cells were pretreated or untreated with emodin (40 μm) for 12 h and TGFβ (5 ng·mL^−1^) was added to the media for 48 h. Photographs were taken using phase contrast microscope. Scale bars = 20 μm. (D) Effect of a CK2 inhibitor on EMT. A549 cells were treated as in (C). Western blot analysis was performed with the indicated antibodies using β‐actin as the loading control for total cell lysates. (E) Effect of CKD on cell morphology. Scale bars = 50 μm. (F) Effect of CKD on EMT. Control and CKD cells were treated with TGFβ for 48 h. Western blot analysis was performed with the indicated antibodies using β‐actin as the loading control for total cell lysates. (G) Effect of CKD on motility of the cells. Migration assays were performed in the presence or absence of TGFβ. The images shown from one experiment are representative of two experiments. Sscale bars = 200 μm.

An increase in CK2 activity by CK2α overexpression induced EMT in the cancer cells (Ko *et al*., 2012) and TGFβ‐induced EMT in A549 cells (Kasai *et al*., 2005). To investigate whether CK2 was activated during TGFβ treatment, A549 cells were treated with TGFβ and harvested at 0, 24, 48 and 96 h after treatment. Lysates were prepared, and an *in vitro* kinase assay and western blot analysis were performed. CK2 activity peaked at 48 h after TGFβ treatment and the E‐to N‐cadherin switch was observed 24 h after TGFβ treatment (Fig. 1A). To examine whether TGFBRI kinase activity was required for the CK2 activation, A549 cells were pretreated with the TGFBRI kinase inhibitor, SB431542. We found that, without TGFBRI activation, neither the increase in CK2 activity, nor the cadherin switch occurred (Fig. 1B). To examine whether the CK2 activation is required for TGFβ‐induced EMT, A549 cells were treated with the pharmacological CK2 inhibitor, emodin, and then with TGFβ for 48 h. In the absence of emodin, A549 cells changed from a rounded, epithelial morphology to a spindle and fibroblast‐like appearance (Fig. 1C) and the E‐ to N‐cadherin switch (Fig. 1D) was observed. However, in the presence of emodin, morphological changes and cadherin switch were not observed (Fig. 1C,D). To confirm these results, we generated stable *CSNK2A1* knockdown (CKD) A549 cells. Previously, we reported that CKD could decrease cellular CK2 activity (Ko *et al*., 2012). We found that CKD cells showed neither morphological changes (Fig. 1E), nor the cadherin switch (Fig. 1F) with TGFβ treatment. To examine the effect of CKD on motility of the cells, migration assays were performed. Even in the presence of TGFβ, CKD cells were not motile (Fig. 1G).

### 3.2 CK2 activation independent on SMAD4

**Figure 2:**
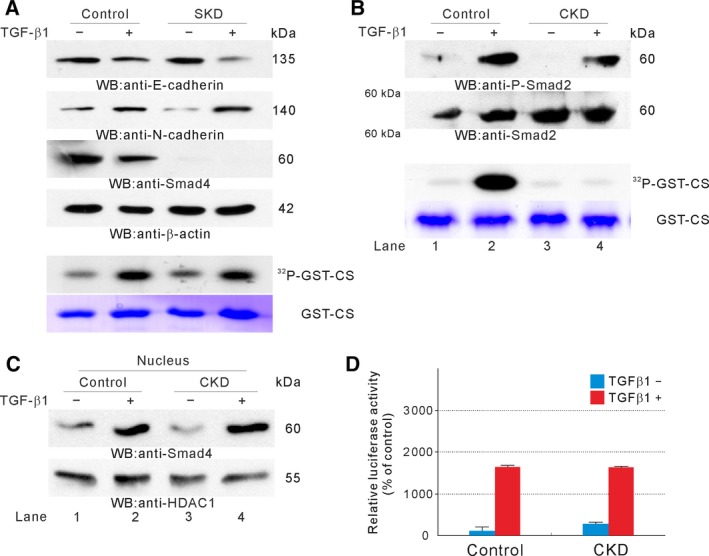
SMAD4 independent CK2 activation. (A) Effect of SKD on CK2 activation. Control or SKD A549 cells were treated, or not, with TGFβ for 48 h. Western blot analysis was performed with the indicated antibodies (top) and an *in vitro* kinase assay was performed (bottom). (B) Effect of CKD on TGFβ‐induced SMAD2 phosphorylation. Control or CKD cells were treated, or not, with TGFβ for 48 h. Western blot analysis was performed with the indicated antibodies (top) and an *in vitro* kinase assay was performed (bottom). (C) Effect of CKD on TGFβ‐induced nuclear localization of SMAD4. Control or CKD cells were treated, or not, with TGFβ for 48 h. Cells were fractionated, and western blot analysis was performed with the indicated antibodies using the nuclear fraction. (D) Effect of CKD on p3TP‐lux‐promoter activation by TGFβ. The luciferase activity of p3TP‐Lux was normalized to that of pRL‐TK. Data represent the mean ± SD of one experiment performed in triplicate. Similar results were obtained from two independent experiments.

Because the increase in CK2 activity depended on TGFBRI kinase activity, we then examined whether canonical SMAD signaling was required for activation. To disrupt canonical SMAD signaling, we generated stable *SMAD4‐*knockdown (SKD) A549 cells using shRNA. When SKD cells were treated with TGFβ, EMT was induced and CK2 was activated (Fig. 2A). Next, we examined whether CK2 was required for SMAD signaling. When CKD cells were treated with TGFβ, SMAD2 was phosphorylated (Fig. 2B, lane 2 vs. lane 4) and SMAD4 was translocated into the nucleus (Fig. 2C, lane 2 vs. lane 4) even in the absence of CK2 activation (Fig. 2B, lane 2 vs. lane 4). There was no difference in p3TP‐Lux (Wrana *et al*., 1992) luciferase activity between the control and CKD cells by TGFβ treatment (Fig. 2D). Collectively, these results suggested that CK2 activation and EMT did not require SMAD4.

### 3.3 CK2β degradation by TGFβ signaling

**Figure 3:**
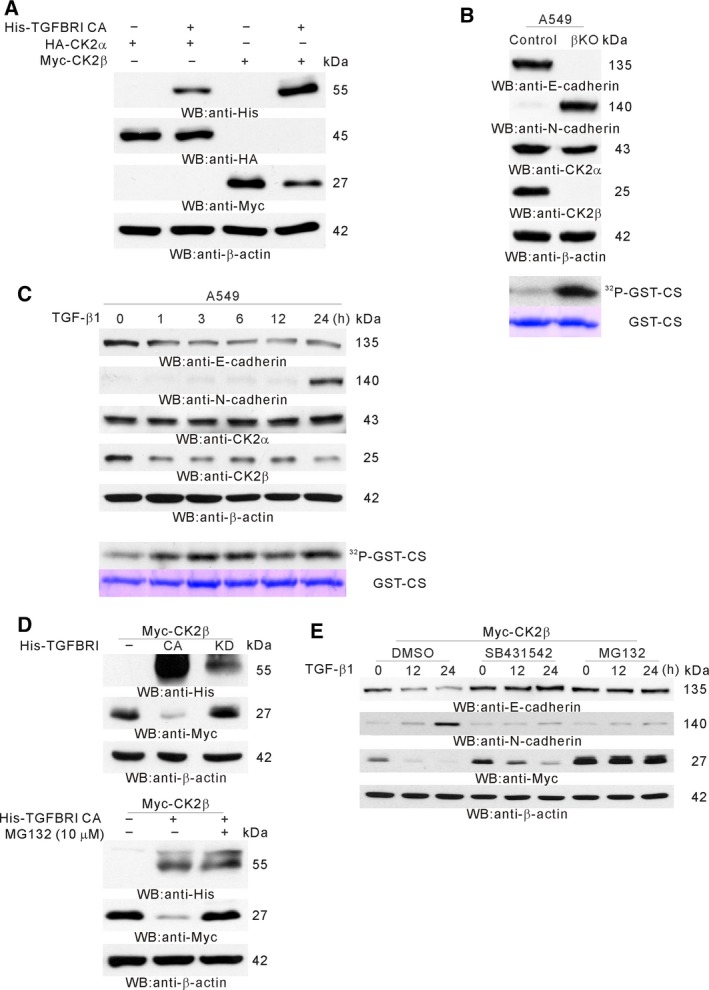
Degradation of CK2β by TGFβ signaling. (A) Effect of TGFβ signaling on the protein level of each CK2 subunit. HEK 293 cells were cotransfected with HA‐CK2α or Myc‐CK2β along with His‐TGFBRI CA. Western blot analysis was performed with the indicated antibodies. (B) Effect of βKO on CK2 activity and EMT. βKO cells were generated using the CRISPR/Cas9 system. Western blot analysis was performed with the indicated antibodies (top) and an *in vitro* kinase assay was performed (bottom). (C) Effect of TGFβ on the protein levels of endogenous CK2 subunits and CK2 activity. A549 cells were treated with TGFβ for the indicated time periods. Western blot analysis was performed with the indicated antibodies (top) and an *in vitro* kinase assay was performed (bottom). (D) TGFBRI kinase activity‐dependent (top) and proteasome‐dependent (bottom) CK2β degradation. HEK 293 cells were cotransfected with His‐TGFBRI CA or His‐TGFBRI KD along with Myc‐CK2β, or cotransfected with His‐TGFBRI CA along with Myc‐CK2β, followed by MG132 (10 μm) treatment. Western blot analysis was performed with the indicated antibodies. (E) TGFBRI kinase activity‐dependent and proteasome‐dependent degradation of CK2β and EMT. A549 cells were transfected with Myc‐CK2β and pretreated with dimethylsulfoxide, SB431542 or MG132 for 12 h. The cells were treated with TGFβ for the indicated time periods. Western blot analysis was performed with the indicated antibodies.

Because TGFβ‐induced CK2 activation depended on TGFBRI kinase activity, TGFBRI CA was used for TGFβ signaling instead of TGFβ treatment (Wieser *et al*., 1995). Because unbalanced protein levels of CK2 subunits may drive EMT (Deshiere *et al*., 2013), we then examined whether TGFβ signaling could alter the protein level of CK2α or CK2β. Western blot analysis using lysates from HEK 293 cells cotransfected with TGFBRI CA and either with CK2α or CK2β showed that CK2β expression was decreased CK2β but did not affect CK2α protein levels (Fig. 3A). To determine the effect of CK2β downregulation on CK2 activity, *CSNK2B*‐knockout (βKO) A549 cells were generated using the CRISPR/Cas9 gene knockout system. Western blot analysis and *in vitro* kinase assay showed that with βKO, the E‐ to N‐cadherin switch was induced (Fig. 3B, top) and CK2 activity was increased (Fig. 3B, bottom) even in the absence of TGFβ treatment. A nigration assay showed that βKO cells became motile even without TGFβ treatment (Fig. S1). To confirm the effect of TGFβ signaling on the protein level of CK2α and CK2β, A549 cells were treated with TGFβ for 1, 3, 6, 12 and 24 h. Western blot analysis confirmed that the protein level of endogenous CK2α was not altered by TGFβ treatment; however, CK2β was decreased by the treatment (Fig. 3C, top). An *in vitro* kinase assay using the same lysates showed that CK2 activity was increased and maintained until 24 h after TGFβ treatment (Fig. 3C, bottom). To examine whether TGFBRI kinase activity is required for the decrease in CK2β expression, TGFBRI kinase dead was used (lysine 232 is replaced with arginine). Unlike TGFBRI CA, TGFBRI kinase dead did not decrease the protein level of CK2β, indicating that the decrease in the CK2β protein level was dependent on TGFBRI kinase activity (Fig. 3D, top). To examine whether CK2β is degraded by the ubiquitin‐dependent proteasome pathway, cells cotransfected with TGFBRI CA and CK2β were treated with MG132; in the presence of MG132, CK2β was not degraded by TGFβ signaling (Fig. 3D, bottom). To confirm these results, A549 cells were pretreated with SB431542 or MG132 before TGFβ treatment. In the absence of SB431542 or MG132, CK2β was rapidly degraded, and the E‐ to N‐cadherin switch was induced (Fig. 3E; dimethylsulfoxide). When SB431542 was pretreated, the CK2β was degraded more slowly than in dimethylsulfoxide treated cells, and the E‐ to N‐cadherin switch was not induced (Fig. 3E; SB431542). When MG132 was pretreated, CK2β was not degraded, and the E‐ to N‐cadherin switch was not induced (Fig. 3E; MG132).

### 3.4 CHIP and WWP1 are E3 ubiquitin ligases for CK2β degradation

**Figure 4:**
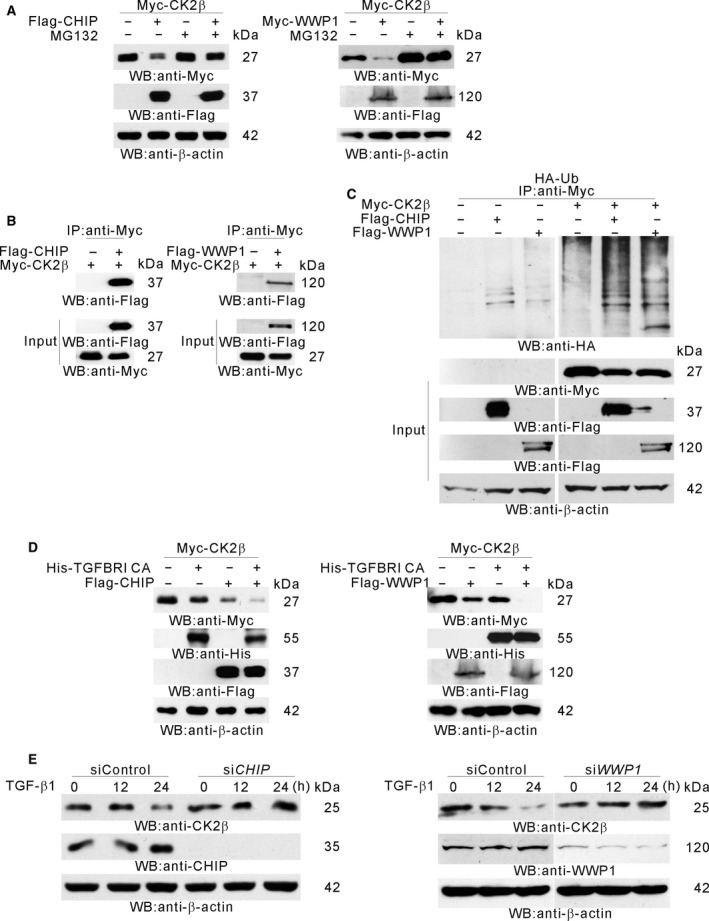
CHIP and WWP1 as E3 ubiquitin ligases for TGFβ‐induced CK2β degradation. (A) CHIP‐ and WWP1‐mediated degradation of CK2β. HEK 293 cells were cotransfected with Flag‐CHIP and Myc‐CK2β (left) or with Flag‐WWP1 and Myc‐CK2β (right) and then treated or not with MG132 for 12 h. Western blot analysis was performed with the indicated antibodies. (B) CK2β interaction with CHIP or WWP1. HEK 293 cells were cotransfected with Flag‐CHIP and Myc‐CK2β (left) or with Flag‐WWP1 and Myc‐CK2β (right). Immunoprecipitation was performed using anti‐Myc Ab followed by western blot analysis. The expression controls were given in the Input. (C) CHIP‐ or WWP1‐induced polyubiquitination of CK2β. HEK 293 cells were cotransfected with indicated plasmids and then treated with MG132 for 12 h. Immunoprecipitation was performed using anti‐Myc Ab. Western blot analysis was performed with anti‐HA Ab. The expression controls were given in the Input. (D) CHIP‐ or WWP1‐mediated CK2β degradation during TGFβ signaling. HEK 293 cells were cotransfected with indicated plasmids. Western blot analysis was performed with the indicated antibodies. (E) CHIP‐ or WWP1‐mediated CK2β degradation in TGFβ signaling. A549 cells were transfected with either siRNA against *CHIP* (left) or siRNA against *WWP1* (right) and then treated with TGFβ for the indicated time periods. Western blot analysis was performed with the indicated antibodies.

Because CK2β is polyubiquitinated in TGFβ signaling (Fig. S2), we examined which E3 ligase (s) is involved in the ubiquitination of CK2β. Among the E3 ubiquitin ligases known to be involved in TGFβ signaling and that we used for screening (De Boeck and ten Dijke, 2012), CHIP and WWP1 lowered the CK2β protein level (Figs S3 and 4A). MG132 protected CK2β from CHIP‐ and WWP1‐mediated degradation (Fig. 4A) and CK2β interacted with these E3 ligases (Fig. 4B). Both CHIP and WWP1 increased CK2β ubiquitination (Fig. 4C) and, together with TGFβ signaling, CHIP and WWP1 efficiently degraded CK2β (Fig. 4D). To examine the effect of *CHIP* or *WWP1* knockdown on the CK2β protein level during TGFβ signaling, siRNA against *CHIP* or *WWP1* was used. The CK2β protein level was not decreased by TGFβ treatment in the absence of either CHIP or WWP1 expression (Fig. 4E).

### 3.5 Dephosphorylation‐dependent CK2β degradation

**Figure 5:**
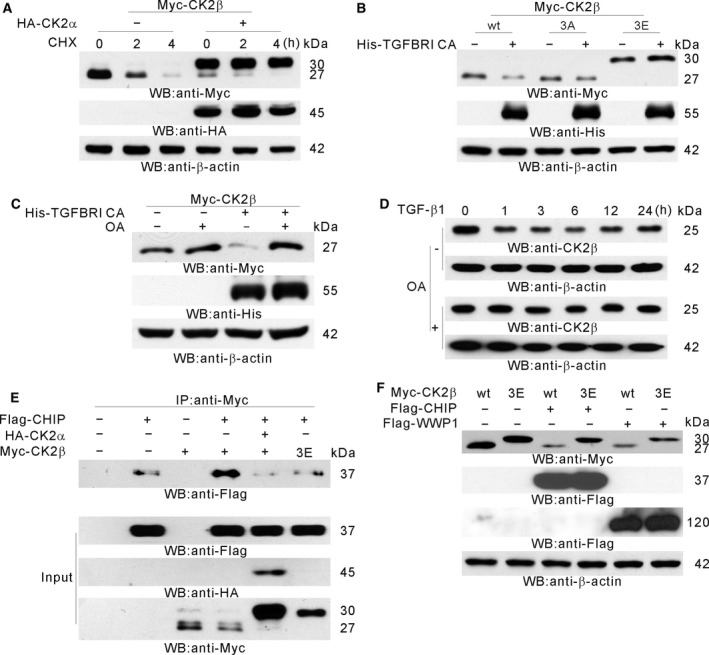
Requirement of dephosphorylation in TGFβ‐induced CK2β degradation. (A) Stabilization of CK2β by CK2α‐mediated autophosphorylation. HEK 293 cells were transfected with or without HA‐CK2α along with Myc‐CK2β and then treated with cycloheximide (CHX) for the indicated time periods. Western blot analysis was performed with the indicated antibodies. (B) Protection of CK2β degradation by phosphorylation. HEK 293 cells were transfected with or without TGFBRI CA along with wt Myc‐CK2β (wt), Myc‐CK2β 3A (3A) or Myc‐CK2β 3E (3E). Western blot analysis was performed with the indicated antibodies. (C) OA protection of CK2β degradation by TGFβ signaling. HEK 293 cells were transfected with or without TGFBRI CA along with Myc‐CK2β and then treated with OA (2 nm) for 12 h. Western blot analysis was performed with the indicated antibodies. (D) OA protection of endogenous CK2β degradation by TGFβ treatment. A549 cells were pretreated with OA for 12 h and then treated with TGFβ for the indicated time periods. Western blot analysis was performed with the indicated antibodies. (E) Preferential binding of CHIP to dephosphorylated CK2β. HEK 293 cells were cotransfected with indicated plasmids. Immunoprecipitation was performed using anti‐Myc Ab followed by western blot analysis using indicated antibodies. The expression controls were given in the Input. (F) Efficient degradation of wt CK2β by CHIP and WWP1. HEK 293 cells were cotransfected with indicated plasmids. Western blot analysis was performed with the indicated antibodies.

As reported previously (Zhang *et al*., 2002), CK2β was autophosphorylated by CK2α and stabilized (Fig. 5A). To examine whether TGFβ signaling could degrade phosphorylated CK2β, phosphomimetic CK2β 3E mutant was used. We found that phosphorylated CK2β was not degraded by TGFβ signaling (Fig. 5B). Based on these results, we assumed that dephosphorylation of CK2β preceded the degradation of CK2β. Because it was reported that TGFβ signaling could activate OA‐sensitive protein phosphatase (Petritsch *et al*., 2000), HEK 293 cells cotransfected with TGFBRI CA and Myc‐CK2β were treated or untreated with 2 nm OA. In the presence of OA, CK2β was no longer degraded by TGFβ signaling, indicating that the degradation required the activation of OA‐sensitive phosphatase (Fig. 5C). To confirm these results, A549 cells were pretreated or untreated with OA for 12 h and then treated with TGFβ for the indicated time periods. Western blot analysis showed that OA treatment protected endogenous CK2β from degradation (Fig. 5D). To examine whether CHIP binds to dephosphorylated CK2β, HEK 293 cells were cotransfected with CHIP and CK2β in the presence or absence of CK2α or CK2β 3E mutant. IP and western blot analysis revealed that CHIP could bind more selectively to unphosphorylated CK2β (Fig. 5E). CHIP and WWP1 efficiently degraded wt CK2β but did not degrade CK2β 3E mutant (Fig. 5F).

## 4 Discussion

The present study shows that TGFβ activated CK2 and activation was required for TGFβ‐induced EMT. We observed that TGFβ signaling decreased the CK2β protein level, thereby resulting in an imbalance between the protein levels of the catalytic α and regulatory β subunits, leading to CK2 activation. This decrease was TGFBRI kinase activity‐dependent and proteasome‐dependent. We also observed that the E3 ubiquitin ligase involved in CK2β degradation was CHIP, and that OA‐sensitive phosphatase‐mediated dephosphorylation was required for CHIP‐mediated degradation.

Although CK2 is known to be a ligand‐independent, constitutively active serine/threonine kinase, EGF could activate CK2 (Ackerman *et al*., 1990; Ji *et al*., 2009). Apart from EGF, TGFβ also could activate CK2 (Fig. 1). Although CK2 activity peaked at 50 min post EGF treatment, and returned to baseline by approximately 120 min (Ackerman *et al*., 1990), CK2 activity peaked approximately at 48 h post TGFβ treatment (Fig. 1) suggesting that EGF and TGFβ might operate with different mechanisms for CK2 activation. Although EGF activated CK2 via ERK2‐mediated CK2α phosphorylation (Ji *et al*., 2009), TGFβ might activate CK2 by inducing an imbalance between the levels of catalytic α and regulatory β subunits through β subunit degradation (Figs 3 and 4). The results of the present study were supported by previous studies reporting that the imbalance between CK2 subunit levels caused by the reduction of β regulatory subunit is linked to increase in molecular target levels related to EMT in tissue samples from breast cancer patients, and that CK2β‐depleted epithelial cells exhibited EMT‐like morphological changes, as well as enhanced migration and anchorage‐independent growth (Deshiere *et al*., 2011, 2013). Although CK2β knockdown could induce EMT phenotye and strongly elevate TGFβ2 expression, blocking the TGFβ signaling pathway did not counteract the EMT phenotype (Deshiere *et al*., 2011). Consistent with these results, we demonstrated that βKO A549 cells showed EMT phenotypes even in the absence of TGFβ treatment (Figs 3B and S1). We also showed that CK2 activation and TGFβ‐induced EMT were blocked by TGFBRI kinase inhibitor (Fig. 1B) and also that EMT was not induced in the absence of CK2 activation (Fig. 1D,F) and CK2β downregulation (Fig. 3E), suggesting that the CK2 activity increase resulting from downregulation of regulatory CK2β subunit is required for TGFβ‐induced EMT. These results suggest that the roles of TGFβ signaling in EMT induction might comprise CK2β degradation‐dependent CK2 activation through a non‐canonical SMAD signaling pathway and thus CK2β depleted cells no longer required TGFβ signaling for EMT induction.

An increase in CK2 activity by the overexpression of CK2α catalytic subunit induced EMT in cancer cells even in the absence of TGFβ‐dependent canonical SMAD signaling (Ko *et al*., 2012), indicating that CK2 activation might be necessary and sufficient to induce EMT. TGFβ induces the expression of EMT‐related transcription factors, such as SNAIL1 or ZEB1 through SMAD3‐dependent transcription (Hoot *et al*., 2008; Postigo, 2003; Vincent *et al*., 2009). The SMAD pathway is a canonical TGFβ signaling pathway and involves receptor‐regulated SMADs (SMAD2 or SMAD3) and a common partner SMAD (SMAD4). Because SMAD4 is a common partner SMAD, SKD could abolish TGFβ‐mediated SMAD signaling by preventing SMAD2 or SMAD3 from forming a complex with SMAD4. In the absence of SMAD4, CK2 was activated and EMT was induced by TGFβ, indicating that SMAD4 was not required for TGFβ‐induced EMT (Fig. 2A). Our results are supported by a previous study reporting that SMAD4 is necessary for TGFβ‐induced cell‐cycle arrest and migration, although it is not in TGFβ‐induced EMT (Levy and Hill, 2005). By contrast to our observations, it was reported that SMAD4 is indispensable for EMT. RNA interference‐mediated *SMAD4* knockdown or expression of a dominant negative SMAD4 mutant resulted in preserved E‐cadherin expression (Deckers *et al*., 2006; Takano *et al*., 2007). Although the involvement of SMAD4 in EMT is controversial, we showed that TGFβ could not induce EMT in A549 CKD cells (Fig. 1E,D) with no alterations in canonical SMAD signaling (Fig. 2B–D). These results suggest that CK2 activation‐dependent downstream signaling events could be dominant over SMAD signaling‐dependent transcriptional induction of EMT‐related transcription factors in TGFβ‐induced EMT. CK2 could stabilize Snail (MacPherson *et al*., 2010) or β‐catenin (Polakis, 2007; Song *et al*., 2003) by phosphorylation. Stabilized and nuclear localized β‐catenin subsequently upregulates Axin2 expression, upregulated Axin2 shuttles GSK3β out from the nucleus, and thus nuclear Snail can be stabilized (Yook *et al*., 2006). Collectively, we argued that CK2β subunit might mainly act as a regulatory subunit and unbalanced expression of CK2 subunits by signaling mediated CK2β depletion could increase intracellular CK2 activity for downstream signaling event such as EMT.

CK2β is ubiquitinated and degraded through a proteasome‐dependent pathway (Zhang *et al*., 2002). In the present study, we report that TGFβ induced the ubiquitination and degradation of CK2β (Fig. 4). Many E3 ubiquitin ligases participate in the ubiquitin‐dependent degradation of molecules involved in TGFβ signaling (De Boeck and ten Dijke, 2012). We screened some of them and observed that the CK2β protein level was decreased by CHIP or WWP1 expression (Fig. S3). CHIP belongs to the group of really interesting new gene (RING) and RING‐related E3 ligases, and it contains a tetratricopeptide repeat domain involved in Hsp70 and Hsp90 association (Ballinger *et al*., 1999). Hsp90 exists as a complex with Hsc70 and the α and β subunits of CK2 (Suttitanamongkol *et al*., 2002). We showed that CHIP interacted with CK2β (Fig. 4B) and that CHIP preferentially bound to dephosphorylated CK2β (Fig. 5E). Unlike β‐transducin repeat‐containing proteins (β‐TrCP), which specifically ubiquitinate phosphorylated substrates (Laney and Hochstrasser, 1999), CHIP does not require post‐translational substrate modification for ubiquitination. WWP1 belongs to the C2‐WW‐Homologous to E6AP C Terminus (HECT) type E3 ubiquitin ligase family (Verdecia *et al*., 2003). We showed that WWP1 interacted with CK2β (Fig. 4B), although we could not detect preferential binding of WWP1 to dephosphorylated CK2β (data not shown). Instead, we observed that both CHIP and WWP1 degraded wt CK2β but did not degrade CK2 β 3E mutant, suggesting that CHIP and WWP1 might preferentially bind to dephosphorylated CK2β. Our results were partially supported by a previous study reporting that dephosphorylation induces the ubiquitination and degradation of FMRP (fragile X mental retardation protein) in dendrites (Nalavadi *et al*., 2012).

In non‐canonical TGFβ signaling, TGFBRI kinase‐dependent activation and interaction of phosphatase 2A with p70‐S6 kinase could result in the dephosphorylation and inactivation of the kinase, thereby inducing G1 arrest (Petritsch *et al*., 2000). OA is a potent, selective inhibitor of protein phosphatases, completely inhibiting PP2A at 1 nm and PP1 at higher concentrations (IC_50_ = 10–15 nm). In the present study, we treated cells with 2 nm OA and thus PP2A could be completely inhibited; however, this might not be the case for PP1. OA treatment protected CK2β from TGFβ‐induced degradation (Fig. 5), suggesting that PP2A was the phosphatase involved in TGFβ‐induced CK2β degradation. However, we could not inhibit TGFβ‐induced CK2β degradation in the *PPP2CA‐*,* PPP2CB‐* or *PPP2R2A‐*knockout A549 cells generated using the CRISPR/Cas9 system (S. Kim & K. Kim, unpublished observation). Further experiments, including the generation of *PPP2CA* and *PPP2CB* double knockout A549 cells, are required to identify the phosphatase involved in TGFβ‐induced CK2β dephosphorylation.

TGFβ is highly expressed in many cancers (Friedman *et al*., 1995; Levy and Hill, 2006; Picon *et al*., 1998). In advanced cancers, TGFβ promotes tumorigenesis via EMT induction, and thus cancer cells become more invasive and metastatic. Sustained TGFβ signaling could induce sustained CK2 activation, eventually resulting in metastasis.

## 5 Conclusions

In summary, the results of the present study show that TGFβ activated CK2 and activation was required for TGFβ‐induced EMT. TGFβ signaling decreased CK2β expression, thereby causing an imbalance between the protein levels of the catalytic α and regulatory β subunits, resulting in CK2 activation. The decrease in CK2β protein level was dependent on TGFBRI kinase activity and the ubiquitin‐proteasome pathway. The E3 ubiquitin ligases responsible for TGFβ‐induced CK2β ubiquitination were CHIP and WWP1. Dephosphorylation of CK2β by OA‐sensitive phosphatase might be required for CK2 activation in TGFβ‐induced EMT. Therefore, CK2 could be a good therapeutic target for inhibiting metastasis in cancers with high CK2 activity.

## Author contributions

SK was responsible for study design, data analysis and interpretation, and writing of the paper. SH and KY were responsible for data collection, wet laboratory experiments and data analysis. KK was responsible for study design, study results, data interpretation and critical revision of the manuscript.

## Supporting information


**Fig. S1**. Effect of CSNK2B knockout (βKO) on motility.

**Fig. S2**. Polyubiquitination of CK2β by TGFβ signaling.

**Fig. S3**. Screening of E3 ubiquitin ligases for CK2β degradation.
