# Supplementary figures and images for: Protein kinase CK2 activation is required for transforming growth factor β‐induced epithelial–mesenchymal transition
Source: Mol Oncol. 2018 Sep 21;12(10):1811–26. doi: 10.1002/1878-0261.12378 (PMC6165993; doi:10.1002/1878-0261.12378)

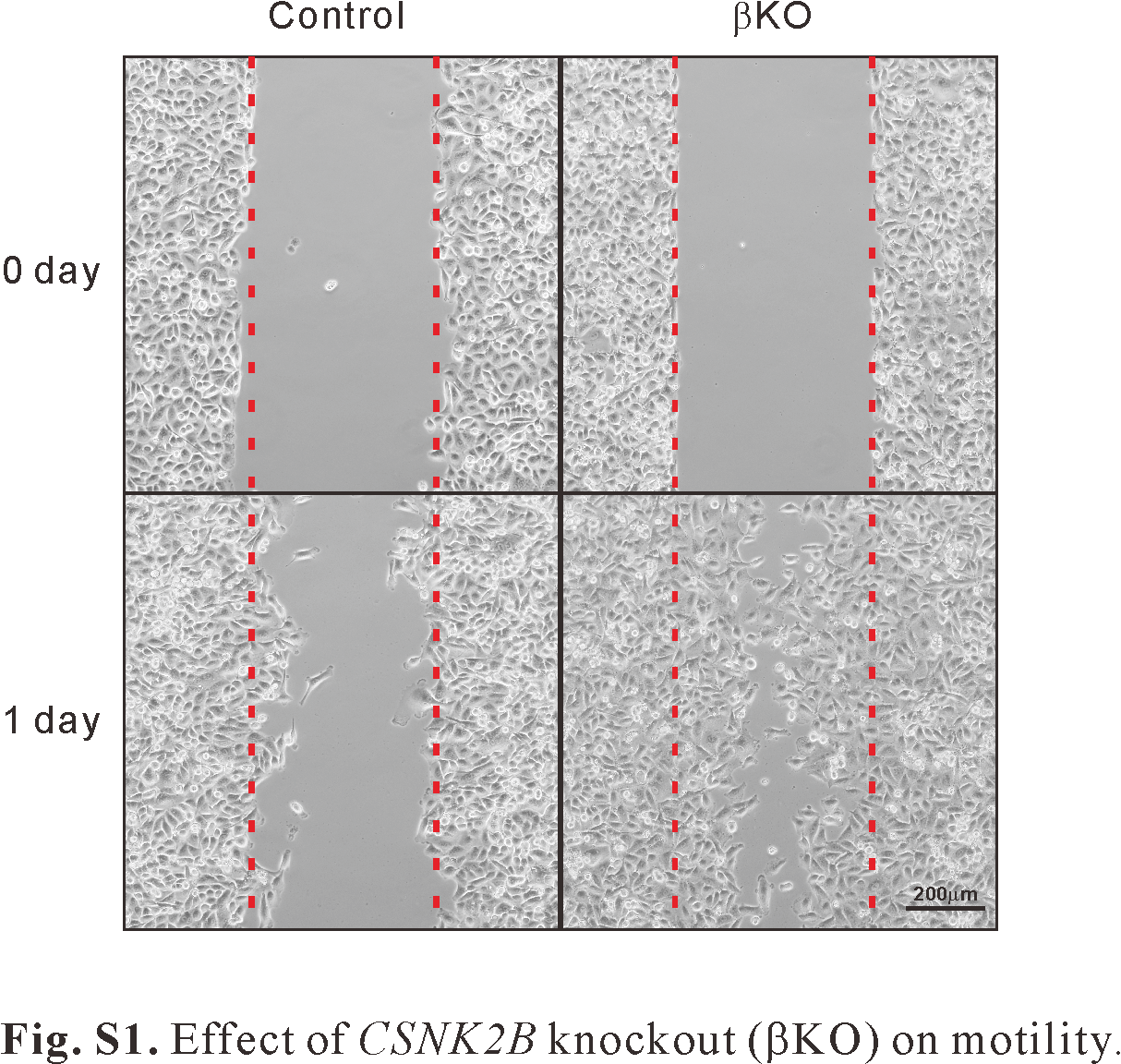

Supplement: Supplementary file 1 — Fig. S1. Effect of CSNK2B knockout (βKO) on motility. [file MOL2-12-1811-s001.tif]

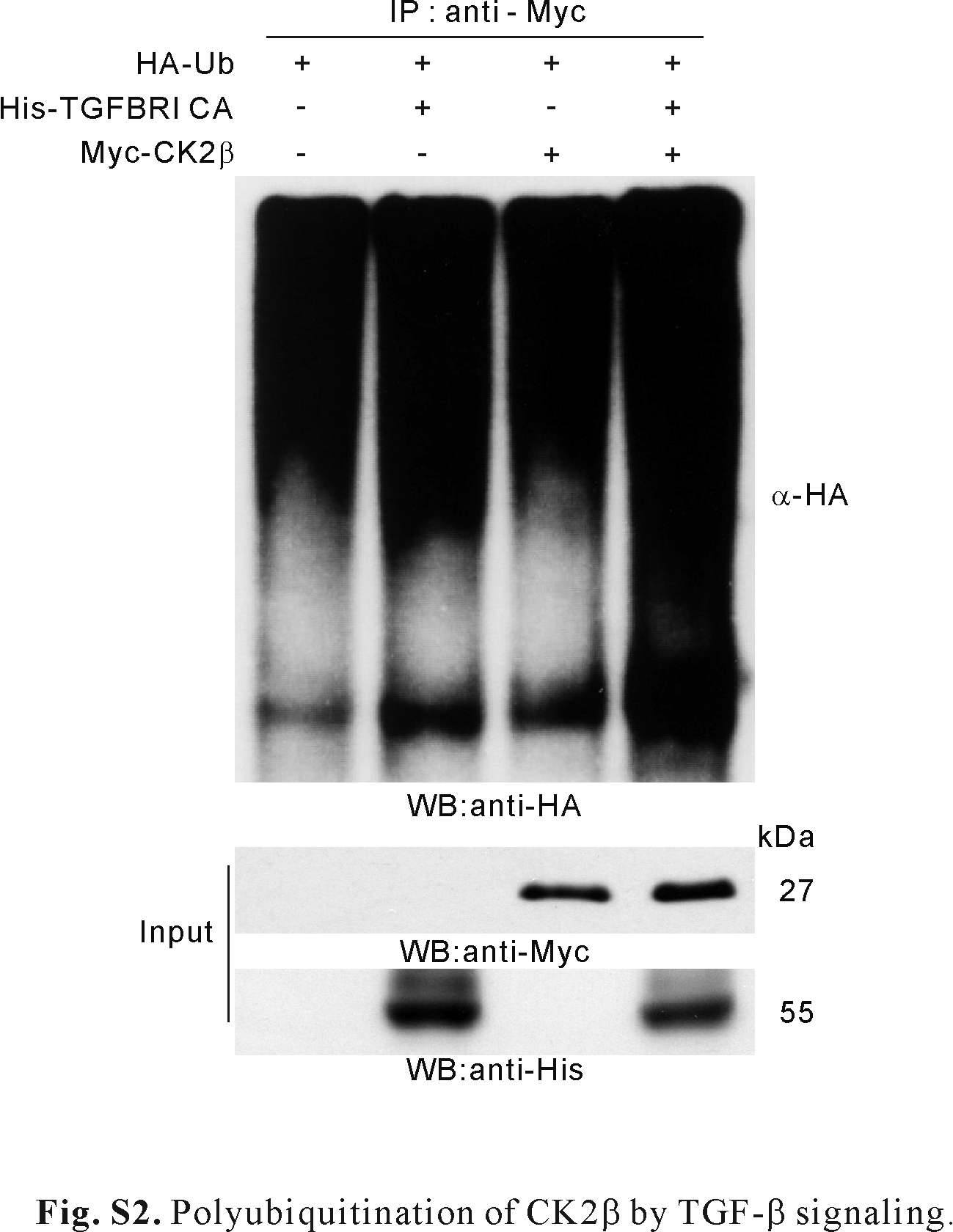

Supplement: Supplementary file 2 — Fig. S2. Polyubiquitination of CK2β by TGFβ signaling. [file MOL2-12-1811-s002.tif]

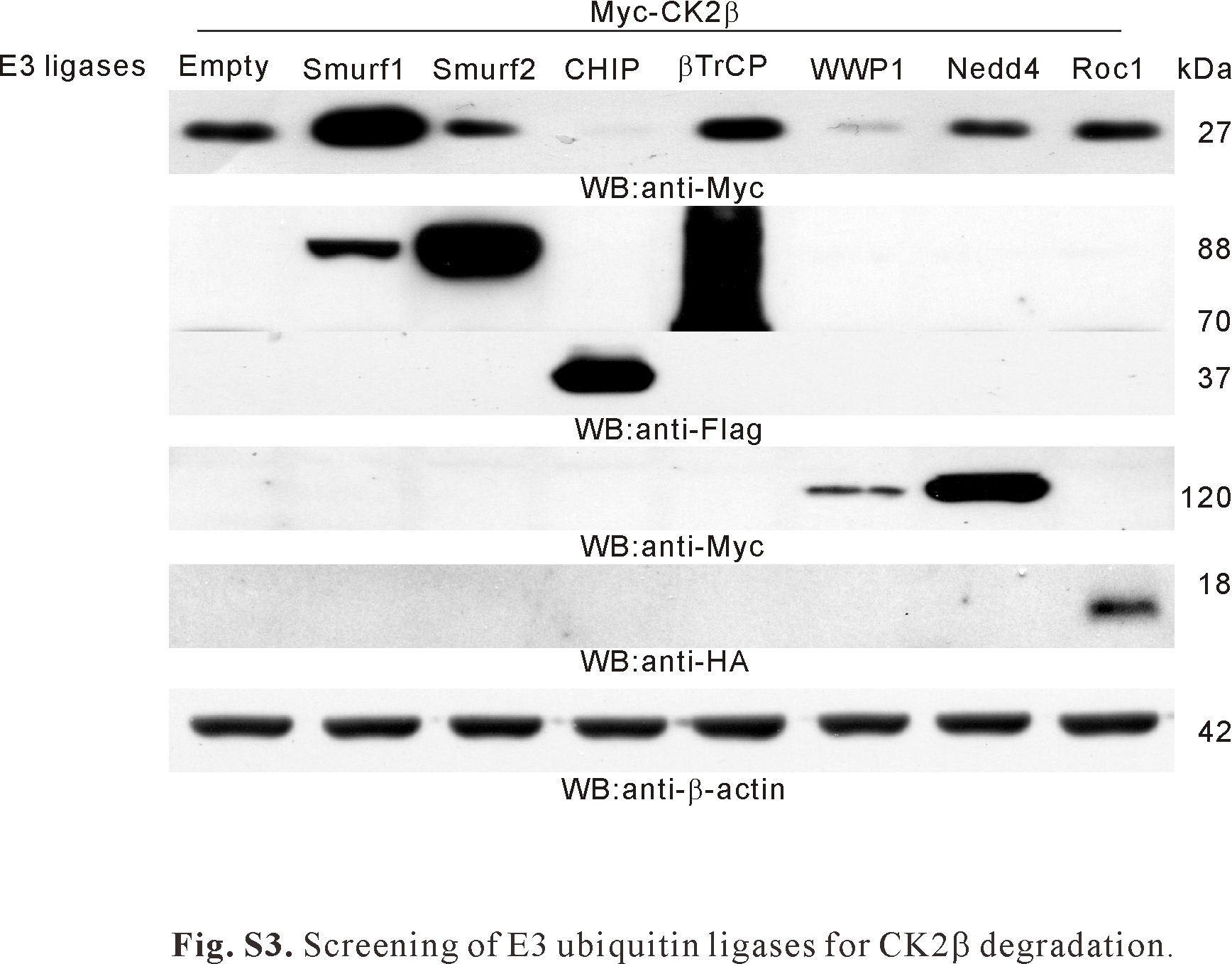

Supplement: Supplementary file 3 — Fig. S3. Screening of E3 ubiquitin ligases for CK2β degradation. [file MOL2-12-1811-s003.tif]
